# Clinical relevance of genetic instability in prostatic cells obtained by prostatic massage in early prostate cancer

**DOI:** 10.1038/sj.bjc.6602311

**Published:** 2005-01-11

**Authors:** R Thuret, K Chantrel-Groussard, A-R Azzouzi, J-M Villette, S Guimard, P Teillac, P Berthon, A Houlgatte, A Latil, O Cussenot

**Affiliations:** 1 CeRePP-EA3104, University Paris 7, France; 2 UroGene®, Génopole, Evry, France; 3 Department of Urology of La Pitié-Salpêtrière Hospital, Paris, France; 4 department of Hormonal Biology, Saint-Louis Hospital, Paris, France; 5 Research Laboratory of Pathology-EA2378, Academic Institute of Hematology, Saint-Louis Hospital, Paris, France; 6 Department of Urology, Saint-Louis Hospital, Paris, France; 7 Department of Urology, Val-de-Grâce Hospital, Paris, France; 8 Department of Urology, Tenon, Paris, France

**Keywords:** biomarker, diagnosis, localised cancer, LOH

## Abstract

We investigated whether genetic lesions such as loss of heterozygosity (LOH) are detected in prostatic cells obtained by prostatic massage during early diagnosis of prostate cancer (CaP) and discussed their clinical relevance. Blood and first urine voided after prostatic massage were collected in 99 patients with total prostate-specific antigen (PSA) between 4 and 10 ng ml^−1^, prior to prostate biopsies. Presence of prostatic cells was confirmed by quantitative RT–PCR analysis of PSA mRNA. Genomic DNA was analysed for LOH on six chromosomal regions. One or more allelic deletions were found in prostatic fluid from 57 patients analysed, of whom 33 (58%) had CaP. Sensitivity and specificity of LOH detection and PSA free to total ratio <15% for positive biopsy were respectively 86.7 and 44% (*P*=0.002) for LOH, and 55 and 74% (*P*=0.006) for PSA ratio <15%. Analysis of LOH obtained from prostatic tumours revealed similar patterns compared to prostatic fluid cells in 86% of cases, confirming its accuracy. The presence of LOH of urinary prostatic cells obtained after prostatic massage is significantly associated with CaP on biopsy and may potentially help to identify a set of patients who are candidates for further prostate biopsies.

Prostate cancer (CaP) is one of the most common cancers in men in Western countries and is second in terms of mortality (Greenlee *et al*, 2000). During the year 2000, in Europe, 189 466 new cases of CaP were diagnosed with 81 353 CaP-related deaths. In order to reduce mortality, prostate cancer should be diagnosed at an early stage when the tumour is still organ confined. Whereas the treatment of advanced CaP is inefficient, an organ-confined tumour can be cured by radical prostatectomy, radiotherapy or cryotherapy.

The early detection of CaP is actually carried out by the association of digital rectal examination (DRE) and serum total prostate-specific antigen (tPSA) level. The usual upper limit for tPSA is 4 ng ml^−1^. However, between 4 and 10 ng ml^−1^, there exists a diagnostic grey zone with an estimated probability of only 40% of CaP in men with normal findings on DRE. Prostrate cancer can only be accurately differentiated from benign prostate hyperplasia (BPH) by pathological proof, usually obtained by prostatic biopsies (PB). To refine the indications for PB, and therefore to reduce morbidity, new indicators based on tPSA were developed: age-adjusted tPSA cutoffs, tPSA velocity or tPSA density where the elevated tPSA is indexed to the gland size (Brawer, 1999). The most commonly used indicator is free/total PSA ratio (f/tPSA), which may help to predict CaP or BPH in its extreme values (Catalona *et al*, 1997). However, the probability of CaP at biopsy among men with a PSA value between 4 and 10 ng ml^−1^ and normal findings on DRE ranged from 56% for men with an f/tPSA ratio of <10% to 8% for men with a ratio >25% (Catalona *et al*, 1998), underlying the lack of accuracy of the f/tPSA indicator. The difficulty of estimating the risk of CaP when there is a suspicious DRE without nodule, when tPSA ranges between 4 and 10 ng ml^−1^ or when f/tPSA has an average value (between 15 and 25%), taken together with the morbidity due to PB, highlights the need of new predictive markers for CaP.

Several authors have used prostatic fluid to develop new diagnostic markers for CaP. Recently, investigators examined the presence of glutathione *S*-transferase (GSTP1) hypermethylation in shed neoplastic cells and urinary debris (Cairns *et al*, 2001). However, its sensitivity (27%) is too low for clinical practice. Telomerase activity was detected after prostatic massage in freshly voided urine (Meid *et al*, 2001), but this approach did not reach clinical practice because of low sensitivity (40%) for the detection of low-grade tumours. The evaluation of ornithine-decarboxylase (ODC) expression in prostatic fluid after prostatic massage has shown an uptranscriptional regulation in CaP (Mohan *et al*, 1999), but intraindividual variations have been observed. Interestingly, the quantification in urine samples after prostatic massage of the CaP-specific gene DD3 (PCA3) transcripts has shown a sensibility and a specificity of respectively 67% and 83% (Hessels *et al*, 2003). Presently, one limit of the clinical acceptance of tests based on quantification of transcripts in urinary sample is the need to preserve RNA quality and to manage immediate frozen sample in clinical pratice. Urinary tests based on DNA sample, like microsatellite analysis or FISH, have already shown their ability to detect bladder carcinoma in clinical practice.

Loss of heterozygosity (LOH) is the most frequent genetic abnormality in CaP. Recurrent LOH in CaP has been found on chromosome arms 7q, 8p, lOq, 12p, 13q, 16q, 17q and 18q, suggesting the presence of tumour suppressor genes (Gao *et al*, 1995; Latil *et al*, 1999; Elo and Visakorpi, 2001). Although these tumour suppressor genes are unknown, we have previously shown accurate detection of CaP by investigating genetic alterations in prostatic cells collected in urinary sediments (Cussenot *et al*, 2001). Furthermore, prognostic significance has been associated with deletions on chromosome 8p22, which predicts disease progression (Matsuyama *et al*, 2001), and on 12pl2–13 and 13q, which predicts aggressiveness (Dong *et al*, 2000; Kibel *et al*, 2000). In order to extend our previous work (Fromont *et al*, 2003), the aim of this study was to develop a new noninvasive method to detect early stages of CaP using LOH analysis of 7q31, 8p22, 12pl3, 13ql4, 16q23.2 and 18q21. We have analysed these six regions in prostatic cells collected in urinary sediments after prostatic massage for LOH, and compared them with the f/tPSA values and results of prostatic biopsy.

## PATIENTS AND METHODS

### Patients’ selection

Samples were collected in two separated centres. Men who presented with suspected CaP requiring PB were included in this study. CaP was suspected with a PSA >4 ng ml^−1^ and/or suspicious DRE. Patients with a tPSA over 10 ng ml^−1^ or with evident clinical T3 CaP at DRE were excluded, as well as men with a history of urothelial tumours. Agreement for this clinical trial was obtained from the ethics committee of Paris-StLouis (CCPPRB No. 2000/69-29/12/2000). Informed consent was obtained from each patient.

A total of 99 patients (mean: 65.4 years; s.d.: 7.5) with a tPSA (mean: 6.7ng ml^−1^; s.d.: 1.76) in the diagnostic grey zone (4–10 ng ml^−1^) were analysed for f/tPSA ratio (mean: 16.1; s.d.: 5.9) and the presence of LOH in prostatic fluid cells. All had, at least sextant, transrectal ultrasound-guided PB.

### Sample collection

Venous blood was collected from each patient for both PSA measurement and DNA extraction. Prostrate-specific antigen measurements were performed in the Saint-Louis hospital department of hormonal biology for every sample with PSA-RIACT and FPSA-RIACT (CIS bio International, Gif-s/-Yvette, France).

Urinary sediments were obtained after prostatic massage performed by DRE. Prostatic massage was performed in order to obtain prostatic secretion at the urethral meat. The first urine voided was collected to extract both RNA and DNA. Urinary samples were centrifuged for 30 min at 4°C (850 **g**), before the cell pellets were immediately stored at −80°C.

In 19 of these cases, formalin-fixed and paraffin-embedded prostatic samples from the patient's radical prostatectomy specimen were also analysed (Table 1 ). In six of these specimens, laser-capture microdissection was used to obtain ‘normal’ prostatic epithelial cells, close to the tumour, for LOH analysis. In each of these samples, prostatic fluid cells and tumour were also analysed (Table 1). Cells were catapulted in 20 *μ*l of 1 × lysis buffer (for 1 ml of lysis buffer: Tris 2 M pH 8 25 *μ*l, EDTA 0.5 M 2 *μ*l, Tween 20 5 *μ*l, proteinase K 50 *μ*l and H_2_O 918 *μ*l) (PALM Micro Laser Technologies, Bernried, Germany).

PCR protocol and genotyping analysis are described below.

### Nucleic acid extractions

All the products used in this study were from SIGMA, (St Louis, MO, USA) unless specified otherwise.

Two distinct methods were used for blood lymphocyte DNA and prostatic cell DNA extraction.

A cell lysis was obtained from blood by red corpuscle lysis solution (Tris 2 M pH 7.6, NaCl 3 M, MgC1_2_ 1 M, H_2_O). After centrifugation (10 min, 24°C, 1200 **g**), a white corpuscle lysis solution (Tris 1 M pH 7, NaCl 3 M, EDTA 0.4 M, SDS 10%, H_2_O) was added to the pellet; proteins were precipitated and pelleted by centrifugation (10 min, 24°C, 1200 **g**) with 10 M ammonium acetate. DNA precipitation was obtained by adding cold isopropanol to the supernatant. After centrifugation (5 min and 1200 **g**), the DNA pellet was rinsed with 70% ethanol and recentrifuged. DNA was suspended in TE 10-1 (Tris 2 M pH 7.6, EDTA 0.4 M, H_2_O) under slow agitation at 42°C for 48 h. DNA concentration was calculated by spectrophotometry (DU 640 B, Beckman, USA) at 260 nm and adjusted to 200 ng *μ*l^−1^.

Prostatic cell DNA was obtained using phenol–acid/chloroform extraction, after proteinase K treatment of the microdissected tumour cells.

RNA extraction was performed only from urinary sediments according to Chomczynski's method (Chomczynski and Sacchi, 1987). RNA was stored at −80°C in DEPC-treated water.

### RNA analysis

To determine the presence of prostatic cells and their proportion compared with other cell types, RNA analysis was performed by quantitative real-time PCR after reverse transcription (RT).

After a first RNA incubation (1 *μ*l of total RNAs in 3 *μ*l DEPC water for 5 min at 57°C), RT was performed with 16 *μ*l of reaction mix (buffer 5 × 4 *μ*l, DTT 0.1 mM 2 *μ*l, DNTP 10 mM each 1 *μ*l, Rhex 6 *μ*l, RNAsin 0.5 *μ*l, Superscript II 0.5 *μ*l and H_2_O 2 *μ*l) by three cycles of incubation: 10 min at 20°C, 30 min at 42°C and 5 min at 99°C (Peltier Thermal Cycler-200, MJ Research, INC Massachussets, USA).

Quantification of prostate-specific mRNA encoding PSA (5′-acc aga gga gtt ctt gac ccc aaa-3′; 5′-ccc cag aat cac ccg agc ag-3′) and transcripts of PPIA (peptidylprolyl isomerase A gene encoding cyclophilin A) gene, used as an endogenous RNA control (5′-gtc aac ccc acc gtg ttc tt -3′; 5′-ctg ctg tct ttg gga cct tgt-3′), was performed by means of the SYBR® Green PCR Core Reagents kit (Applied Biosystems, Palo Alto, CA, USA) using an ABI Prism 7700 Sequence Detection System (Applied Biosystems, Palo Alto, CA, USA). To determine which samples had a sufficient proportion of prostatic cells for genetic analysis, we ensured that the PPIA and PSA mRNA levels corresponded to a straight line (Ct PPIA=0.386 Ct PSA+13.82) (data not shown). In total, 82 samples contained a satisfactory proportion of prostatic cells for genotyping analysis.

### Genotyping analysis

Extracted DNA from blood and prostatic cells was amplified by PCR, using 14 microsatellite markers localised on chromosome arms 7q31, 8p22, 12pl3, 13ql4, 16q23.2. and 18q21: D7S480, D7S522, D7S523, D8S261, D8S1786, D12S77, D12S358, D13S153, D13S272, D13S284, D16S507, D16S518 (Dib *et al*, 1996), and D18S39, D18S46 (Straub *et al*, 1993). PCR amplification was carried out with 3 ng of DNA in a 20 *μ*l final volume of reaction mix (dNTP 0.25 mM, Tris 1 M, boric acid 0.9 M, EDTA 0.01 M, 20 pmol of each primer (MWG Biotech, Ebersberg, Germany), 0.75 *μ*l of DMSO, Taq polymerase 1 U (Qbiogen, Illkirch, France)). The PCR protocol was previously described (Berthon *et al*, 1998) and annealing temperature was optimised for each primer.

Then, 1 *μ*l of PCR product was added to 1 *μ*l of blue dextran and 3 *μ*l of formamide. After a 2 min denaturation at 94°C, amplified fragments were analysed on an automatic sequencer (377 DNA sequencer, Applied Biosystems, Palo Alto, CA, USA) by electrophoresis in TBE buffer (Tris 0.089 M, borate 0.089 M, EDTA 0.002 M pH 8) through a 4% polyacrylamide (Acryl/Bisacryl 29/1) 6 M urea gel. Analysis was performed by GeneScan 3.1 Fragment Analysis Software (Applied Biosystems, Palo Alto, CA).

Loss of heterozygosity was considered to be present when the relative intensity of the two alleles in urinary sample DNA differed from the relative intensity in leuckocyte DNA by a factor of at least 1.3. This threshold, which is suitable for tumour LOH detection (Zenklusen *et al*, 1994), can also be used for the urinary sediments of this study because of the quality selection criteria applied on the samples (see RNA analysis). Each LOH analysis was performed at least twice (repeated PCR amplification, gel separation, and quantification).

### Statistical analysis

All statistical analyses were performed by using the χ^2^ test with the SAS software (version 6.12;. SAS Institute Inc., Cary, NC, USA). All *P*-values were determined by two-sided tests. *P*-values less than 0.05 were considered to be statistically significant.

## RESULTS

Out of 99 patients included in this study, a set of 81 patients had a sufficient proportion of prostatic cells in urinary sediment to perform LOH analysis, of which 38 had CaP on PB (Tables 1 and 2 ). The distribution of age, tPSA and f/tPSA ratio in the group with and without positive biopsy were: negative biopsy: age 65.2 s.d. 5.8; tPSA 6.7 s.d. 1.8; f/tPSA 18.2 s.d. 6.0; positive biopsy: age 66.5 s.d. 9.2; tPSA 6.7 s.d. 1.7; f/tPSA 13.8 s.d. 5.3. Allelic deletions were founded for 57 patients, of whom 33 (58%) had CaP. Within this group, LOH detection had an 87% sensibility and 44% specificity (Table 1).

Then, we checked the validity of the LOH test in urinary sediments by comparing them with the LOH of the corresponding tumours in 19 patients (Table 1). We defined three classes of results: strictly identical results with the same patterns of LOH in tumour and urinary sediments; concordant results in which identical patterns were seen in the two samples except for additional tumour LOH; discordant results corresponded to LOH observed in urinary sediment but not in the tumour. The comparison between the two samples has shown that identical LOH was present in 70.6% and concordant results in 86%. Only 14% of the results were discordant (28.1% of them concerned the 16q23.2 region markers). We investigated also the hypothesis that genetic alterations could exist prior to a morphological abnormality. Therefore, the genetic status of histologically ‘normal’ prostatic cells located near the tumour was analysed. In total, 53.3% of allelic deletions observed in tumour DNA were present in the histologically normal prostatic cells (Table 1).

Genetic diagnosis using LOH has been compared to f/tPSA ratios of <25, <20 and <15%, which are considered as the best indicators of CaP (Table 2). Only seven patients (8.6%) had an f/tPSA ratio over two 5%, two of which had CaP. Using the <15% cutoff, the f/tPSA ratio had a sensitivity of 50% and specificity of 77%, which discriminates significantly (*P*=0.006) the group with CaP (Table 3 ). All cancers could be detected if f/tPSA <15% or LOH was used, and in these conditions the specificity was 32%.

Out of the 81 patients (of whom five had a CaP), 25 did not have any LOH detected. In each case, they had an f/tPSA threshold <15%. Of the 81 patients, 57 had one or more LOH, of which 31 had CaP. None of these patients had LOH in all six chromosomal regions. The frequency of 1–3 chromosomal LOHs was not significantly related to the presence of CaP. Of the patients, 56 and 61% respectively had positive biopsies among patients with (one or two) LOH and over three altered regions.

## DISCUSSION

By this clinical trial, using early CaP, we have shown that LOH can be found in cells obtained from urine by prostatic massage. In particular, LOH can be found at hotspot loci associated with CaP, in patients with positive PB.

Assuming that a positive biopsy is the gold standard, the statistical parameters of the CaP detection by the LOH analysis showed 86.8% sensitivity for CaP detection. The fact that the specificity was only 44% (24 false positives) might be due, in part, to the low sensitivity of PB, which is currently estimated between 66 and 85%, and should be close to 83.3% for a CaP with a volume greater than 2 cm^3^ (Terris, 1999). This sensitivity varies according to the location of CaP inside the prostate (71.4% for the peripheral zone cancers and 33.3% for the transition zone cancers). Furthermore, the sensitivity increases by repeated biopsies, underlying the fact that some CaP are missed by the first set of biopsies. With reference to the experience of LOH detection in urine during the follow-up of bladder cancer, the specificity should improve with the patients’ clinical follow-up.

One of the limitations of the study was the small number of prostate specimens available, as only 19 patients out of 99 underwent a radical prostatectomy. Therefore, the clinical significance of the detected tumours according to tumour volume, Gleason score and presence of adverse pathological patterns such as presence of extracapsular extension and seminal vesicle invasion was unknown.

The presence of genetic alterations in ‘normal’ cells located near the tumour implies that this test may be useful to detect CaP at a very early stage or to identify those patients at high risk of developing CaP. Although the roles of multifocality (multiple foci of carcinoma with independent origins) and clonality development (multiple foci of carcinoma in the same prostate derived from a single progenitor) in CaP are still not fully understood, only 11% of LOH in normal tissue could not be found in the adjacent tumour, suggesting a monoclonal expansion of cancerous cells. Hügel and Wernert (1999) and Macintosh *et al* (1998) analysed LOH in different areas of prostate tumour separated by microdissection. They found that a significant proportion of CaP were made of several histological patterns. However, the clonal or multifocal origin of these patterns was not fully elucidated. Whereas each tumour has its own pattern of LOH, different combinations of genetic events could result in similar malignant phenotypes of CaP.

The sensitivity (87%) of LOH detection for CaP was higher than that observed for the detection of telomerase activity (40%) by Meid *et al* (2001) or for the detection of GSTPl hypermethylation (27%) by Cairns *et al* (2001) or quantification of DD3 PCA3 transcripts (67%) (Hessels *et al*, 2003).

Some ‘false-negative’ patients had noninformative loci, because of homozygosity for the marker. This lack of genetic information affected the sensitivity and specificity of LOH analysis. We analysed urinary samples for LOH at six different loci known to be frequently altered in prostate tumours, but other chromosomal regions like 16q22.1 have also been shown to be involved in CaP genesis. The large majority of all molecular mechanisms explaining prostate cancer development are still expected to be discovered.

In order to select those patients likely to have CaP, we have used a noninvasive test based on LOH detection in cells from prostatic fluid. Furthermore, besides diagnosis value, LOH detection could bring prognostic information on CaP aggressiveness. A significant correlation would exist between the presence of certain LOH and relapse after radical prostatectomy. Current follow-up analysis is under way.

## Figures and Tables

**Table 1:** Results of the 14 loci tested on desquaming prostatic cell DNA according to PB results and in comparision with LOH in tumoral and nontumoral DNA cells from prostate specimens

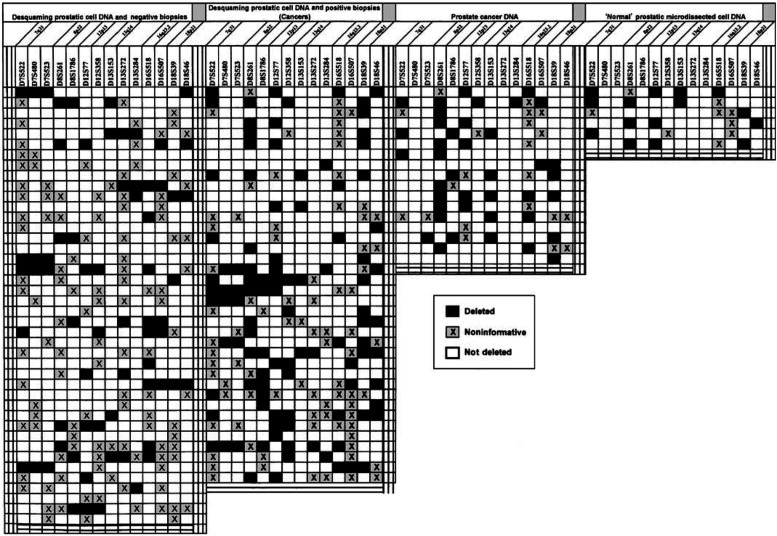

**Table 2:** Pathological patterns of cancers on biopsies and markers (tPSA, f/tPSA, number of LOH)

**Age**	**tPSA**	**f/tPSA**	**Number of LOH**	**Gleason score from biopsy**	**Grade 1**	**Grade 2**	**Stage pT^a^ from biopsy**
73	6	7	1	5	3	2	2a
84	8	20	2	7	4	3	2b
56	6	19	2	4	2	2	2a
63	7	15	2	6	3	3	2a
66	4	15	2	6	3	3	2a
58	6	8	2	6	3	3	2a
69	10	11	3	6	3	3	2b
81	7	11	1	6	3	3	2a
72	7	7	1	6	3	3	2a
75	6	16	1	6	3	3	2a
58	7	6	0	7	4	3	2b
61	8	27	1	4	2	2	2a
64	4	22	5	6	3	3	2a
68	7	7	1	6	3	3	2b
56	7	17	5	4	2	2	2a
57	6	25	5	6	3	3	2a
66	5	17	2	5	3	2	2a
59	8	15	3	4	2	2	2a
75	8	15	0	5	2	3	2b
72	6	14	1	5	2	3	2a
69	6	17	3	5	2	3	2a
73	9	10	2	8	4	4	2a
67	5	15	1	7	3	4	2b
56	6	10	2	7	4	3	2b
70	8	12	3	6	3	3	2a
56	6	7	2	6	3	3	2a
67	5	17	3	6	3	3	2a
93	5	8	1	8	4	4	2b
73	7	16	3	6	3	3	2a
66	4	15	4	6	3	3	2a
60	6	24	1	5	3	2	2a
58	6	17	1	7	4	3	2b
58	7	14	0	6	3	3	2a
70	8	13	3	7	4	3	2c
81	10	12	0	7	4	3	2c
69	8	13	2	6	3	3	2c
49	9	8	0	7	3	4	2c
59	10	8	2	5	2	3	2c

a pT2a=Positive biopsies in one prostatic lobe, less than half-lobe, pT2b=positive biopsies in one prostatic lobe, more than half-lobe, pT2c=positive biopsies in the two prostatic lobes (according to UICC, TNM 2002).

**Table 3:** Diagnostic parameters of the genetic test (LOH) and f/tPSA ratios (threshold values: 15, 20 and 25 %) for 81 patients with a total PSA between 4 and 10 ng ml^−1^

	**Sensitivity %**	**Specificity %**	**χ^2^ (*P*-value)**
Genetic test (LOH)	87	44	*P*=0.002
f/tPSA <25%	95	12	*P*=0.309
f/tPSA <20%	86	33	*P*=0.09
f/tPSA <15%	55	74	*P*=0.006
LOH or f/tPSA <15%	100	32	*P*=0.06
